# 10-Day structured initiation protocol from multiple daily injection to hybrid closed-loop system in children and adolescents with type 1 diabetes

**DOI:** 10.1007/s00592-019-01472-w

**Published:** 2020-01-17

**Authors:** Goran Petrovski, Fawziya Al Khalaf, Judith Campbell, Hannah Fisher, Fareeda Umer, Khalid Hussain

**Affiliations:** Division of Endocrinology and Diabetes, Department of Pediatric Medicine, Sidra Medicine, HB 6E 219, Al Luqta Street, Education City North Campus, PO Box 26999, Doha, Qatar

**Keywords:** Hybrid closed-loop system, Multiple daily injection, Protocol, Type 1 diabetes

## Abstract

**Aim:**

The aim of this study was to evaluate the 10-day initiation protocol for MiniMed 670G hybrid closed-loop (HCL) system in individuals with type 1 diabetes on multiple daily injection (MDI) in achieving desirable glycemic control.

**Methods:**

An open-label single-arm, single-center, clinical investigation in children aged 7–18 years on MDI following a structured protocol: 2 days, HCL system assessment; 5 days, HCL system training (2-h sessions on 5 consecutive days with groups of 3–5 participants and families); 3 days, Manual Mode use of HCL system; 84 days, Auto Mode use of the HCL system, cumulating in 10 days from MDI to Auto Mode activation.

**Results:**

A total of 30 children (age 10.24 ± 2.6 years) were enrolled in the study, and all completed the planned 84 days on Auto Mode. The participants used the sensor for a median of 92% of the time and spent a median of 89% in Auto Mode. The mean HbA1c decreased from 8.2 ± 1.4% (66 ± 15.3 mmol/mol) at baseline to 6.7 ± 0.5% (50 ± 5.5 mmol/mol) at the end of the study (*p *= 0.017). Time in range (70–180 mg/dL) increased from 46.9 ± 18.5% at baseline to 75.6 ± 6.9% in Auto Mode (*p *< 0.001). This was achieved while spending 2.8% of the time below 70 mg/dL and without any severe hypoglycemia or DKA.

**Conclusion:**

Children and adolescents with type 1 diabetes on MDI therapy can successfully initiate the HCL system, using a concise structured 10-day protocol.

## Introduction

Improving glycemic control in individuals with type 1 diabetes without increasing risk of hypoglycemia is a challenge for both individuals and health providers. Intensive insulin treatment in combination with regular self-monitoring of blood glucose (SMBG) is the standard of care for individuals with type 1 diabetes. Nevertheless, the majority of individuals fail to achieve optimal glycemic control with HbA1c below 7% (53 mmol/mol) [1]. Monitoring glucose levels from the interstitial fluid, either continuously with real-time continuous glucose monitoring (CGM) or intermittently, by scanned continuous glucose monitoring (isCGM), demonstrated better diabetes management for children and adolescents with diabetes [2].

Recent technological advances in diabetes treatment have integrated continuous subcutaneous insulin delivery (CSII) with CGM, where insulin delivery can be automated by sensor glucose (SG)-driven algorithms. Insulin delivery suspension when reaching a low glucose level [3, 4] or in prediction of a low glucose level [5, 6] has demonstrated significant reduction in hypoglycemia exposure.

Further development in technology of integrated closed-loop systems provides algorithm-derived automated adjustment of insulin delivery to address both hypoglycemia and hyperglycemia [7–11].

The first approved hybrid closed-loop (HCL) system [12] is the MiniMed 670G system (Medtronic Diabetes, Northridge, CA, USA) for children above 7 years old [13], adolescents and adults with type 1 diabetes. The system [14] uses an algorithm to adjust basal insulin delivery automatically every 5 min based on SG values [15, 16]. Several studies have shown improved HbA1c, time in target range, and SG variability in children [17], adolescents and adults [18, 19] with type 1 diabetes.

Studies in MiniMed 670G system [17, 19] included participants experienced with using CSII therapy, assuming that the success of HCL systems depends on prior use of technology. There is no evidence of the glycemic control achieved by patients on multiple daily injection (MDI) transitioning to the HCL system. Although a recent publication suggested a training and education program for HCL system in patients naïve to insulin pump therapy [20], there is no standardized protocol to initiate HCL system in individuals on MDI.

The objective of this study was to evaluate whether the use of a 10-day structured initiation protocol for MiniMed 670G HCL system in individuals with type 1 diabetes on MDI therapy will provide similar outcomes to the MiniMed 670G pivotal studies, that included individuals on pumps therapy and not patients on MDI, as the latter are the majority of individuals with type 1 diabetes [17, 19].

## Methods

This open-label, non-randomized, single-arm, single-center, clinical investigation study was conducted at Sidra Medicine in Doha, Qatar, and enrolled individuals aged 7–18 years with type 1 diabetes > 1 year, on MDI with SMBG, with or without RT-CGM or isCGM, with no prior pump experience, and with an HbA1c level < 12.5%. Individuals were recruited at regular clinic visits, following the clinical pathway for therapy [21]. This clinical assessment emphasizes the following individual aspects: frequency of SMBG controls per day, knowledge of carbohydrate counting, insulin dose adjustments based on carbohydrate intake and blood glucose level, the ability to identify changes in insulin requirements due to physical activity and sick days, ability to recognize, troubleshoot and manage hyperglycemia and hypoglycemia. Individuals with self-funding capability and those with insurance coverage were recruited in the study.

The initiation protocol consisted of four main stages: HCL system compatibility assessment, HCL system training, Manual Mode and Auto Mode stages.

### Step 1: HCL system compatibility assessment

Interested individuals attended two introduction sessions of 1 h (groups of 8–12), where the MiniMed 670G system was described. Individuals’ responsibilities (bolusing before meal, calibrating the system 3–4 times per day, responding to alerts and alarms, downloading pump data from home) and expectations (improvements in glycemic control, less glucose variability, hypoglycemia minimization) were discussed.

### Step 2: HCL system training

Three-to-five individuals and their parents/guardians attended group training program. The program included five sessions of 2 h on five consecutive days: Day 1—pump buttons and menus, understanding CGM graph, education in sensor calibration and sensor insertion; Day 2—basic training of the different pump modes: Manual Mode, bolus wizard use, basal rates, Auto Mode, introducing the concepts of safe basal; Day 3—practical training on infusion set and reservoir change, Medtronic CareLink Software downloads and creation of personal accounts; Day 4—hypoglycemia, hyperglycemia, exercise and travel management, guidance on the use of temporary target; Day 5—explaining Auto Mode readiness screen, instructing on meal bolus (timing and carb counting refresher), correction bolus, system exits and final evaluation of participants’ ability to handle the HCL system.

Each session was provided by two educators from 12 pm to 2 pm. CGM was initiated on the first day of the training, for education and observational purposes and for baseline data collection (no insulin delivery by the pump). Timing of the long acting insulin injection was moved 2 h ahead each day during training sessions, to reach 12 pm the day before the HCL system was initiated in Manual Mode, to avoid the use of temporary basal at insulin pump initiation.

### Step 3: Manual mode

Participants initiated the use of the HCL system in Manual Mode with suspend before low feature for 72 h to allow the algorithm to collect insulin utilization and CGM data to establish personalized Auto Mode initiation parameters. Sidra’s validated protocol for SAP initiation, as previously described [22], with review of 1-week CGM data (step 2) was used: In short, the protocol inputs the current insulin program (MDI) and calculates a 10–20% reduction in total daily dose, with a 40/60 basal/bolus distribution in four or five basal rates. Insulin-to-carbohydrate ratio (ICR) settings utilize the formula of 300–450/total daily dose (TDD) and the formula of 90–110/TDD (mmol/L) with two CF settings; the nighttime CF factor is set 10–20% higher than the daytime CF. Active insulin is set time (3 h); suspend before low feature is turned on with a threshold of 3.0–3.8 mmol/L (55–70 mg/dL), and glucose target ranges from 5.0 to 7.2 mmol/L (90–130 mg/dL).

### Step 4: Auto mode

The Auto Mode feature of the HCL system was activated 72 h after Manual Mode initiation and used continuously for 84 days. Follow-up visits were scheduled as follows: in clinic on days 3, 7 and 84; and phone call visits on days 14, 28 and 56 after enabling Auto Mode. Participants uploaded data from the pump to CareLink Personal software (Medtronic, Northridge, CA, USA) every 2 weeks.

HbA1c was obtained using point of care DCA Vantage Analyzer (Siemens, Erlangen, Germany) at baseline and at the end of the study.

The study was approved by local and National Ethics Committee in Qatar, and all participants and their guardians signed an informed consent document.

### Statistical analysis

Analysis was performed for the entire study population. Sub-analysis for prior CGM users versus users without prior CGM was predefined. Prior CGM use was defined as a use of Dexcom G5 (Dexcom, San Diego, CA, USA), Guardian Connect (Medtronic, Northridge, CA, USA) or Freestyle libre (Abbott Diabetes Care, Berkshire, UK) at baseline, in combination with SMBG and MDI.

Insulin dosing information and CGM data were collected from CareLink Therapy Management Software during the study. All data are presented as mean ± SD, median, interquartile or as a percentage. The paired student *t* test or paired Wilcoxon test, in case of non-normality, was used in the study. A value of 0.05 was considered statistically significant. Statistical analyses were performed using Statistica 12 (Stat Soft, Tulsa, USA).

## Results

Thirty-eight individuals met the criteria for HCL system use, of these 30 children (age 10.24 ± 2.6 years) consented to initiate the system and were enrolled in the study. The 10-day initiation protocol was implemented in 30 participants, and they all completed the planned 84 days on Auto Mode. Baseline characteristics are shown in Table 1.

**Table 1:** Study participant’s characteristics at baseline

	Participants *N* = 30
Age, years	10.24 ± 2.6
Male, *n* (%)	15 (50%)
Female, *n* (%)	15 (50%)
Weight, kg	38.2 ± 12.5
BMI, kg/m^2^	18.6 ± 3.4
BMI, *z*-score	0.2 ± 0.9
Duration of diabetes, years	2.8 ± 1.7
TDD, U/(kg/day)	0.8 ± 0.3
HbA1c, %	8.2 ± 1.4
HbA1c, mmol/mol	66 ± 15.3
Sensor use, *n* (%)	
RT-CGM	6 (20%)
isCGM	10 (33%)
No sensor	14 (47%)

All values are shown as mean ± SD, except for gender and sensor use

*BMI* body mass index, *TDD* total daily dose of insulin, *SD* standard deviation, *RT-CGM* real-time continuous glucose monitoring (Dexcom G5/Guardian Connect), *isCGM* intermittent continuous glucose monitoring (freestyle libre)

After activating Auto Mode, the participants used the sensor for a median of 92% of the time and spent a median of 89% in Auto Mode at the end of the study.

HbA1c decreased from 8.2 ± 1.4% (66 ± 15.3 mmol/mol) at baseline, to 6.7 ± 0.5% (50 ± 5.5 mmol/mol) at the end of the study (*p *= 0.017). Time in range (TIR) (70–180 mg/dL) increased from 46.9 ± 18.5% at baseline to 74.8 ± 7.1% in Auto Mode (*p *< 0.001). Time below range did not change, while time above range decreased significantly (Table 2). Mean SG decreased from 193 ± 41 to 142 ± 12 mg/dL (*p *= 0.001). TDD and the percentage of basal insulin delivered increased by a mean of 0.1 Unit/kg (*p* = 0.02) during the study, compared to the baseline (Table 2).

**Table 2:** Glucose control, HbA1c, insulin delivered during baseline and study phase

	Baseline	Study	*p*
HbA1c (%)	8.2 ± 1.4 (8.3, 7.1–9.2)	6.7 ± 0.5 (6.7, 6.2–6.9)	0.017
HbA1c (mmol/mol)	66 ± 15.3 (67, 54–77)	50 ± 5.5 (50, 44–52)	0.017
Sensor glucose (mg/dL)	193 ± 41 (191, 158–227)	142 ± 12 (141, 132–148)	0.001
Percent of sensor glucose values in range			
≤ 50 mg/dL	0.4 ± 0.7 (0.2, 0.1–0.6)	0.3 ± 0.5 (0.2, 0.1–0.5)	0.330
51–70 mg/dL	2.7 ± 3.7 (2.5, 1.2–3.4)	2.5 ± 1.8 (2.4, 1.2–2.2)	0.507
71–180 mg/dL	46.9 ± 18.5 (46.5, 33–61)	74.8 ± 7.1 (75.1, 71.0–82.7)	0.001
181–250 mg/dL	25.8 ± 8.8 (25.2, 22.1–32.4)	16.9 ± 5.7 (16.8, 12.1–20.7)	0.001
≥ 251 mg/dL	24.2 ± 16.9 (24.3, 9.2–38.4)	5.5 ± 3.6 (4.9, 2.2–8.2)	0.001
TDD, U/(kg/day)	0.8 ± 0.3 (0.8, 0.6–1.0)	0.9 ± 0.2 (0.8, 0.7–0.9)	0.020
Basal insulin, as % of TDD	36.5 ± 7.2 (37.8, 30,3–40.2)	42.2 ± 6.7 (42.0, 38.4–46.3)	0.037
Weight (kg)	38.3 ± 12.5 (38.6, 26.5–48.4)	39.4 ± 8.9 (38.9, 27.5–49.1)	0.636

Baseline consisted of one week on MDI with CGM; study phase consisted of 12 weeks on Auto Mode. All values are shown as mean ± SD (median, interquartile range)

*MDI* multiple daily injections, *SG*, sensor glucose, *TDD* total daily dose of insulin

### Time in ranges evolution over time

**Fig. 1:**
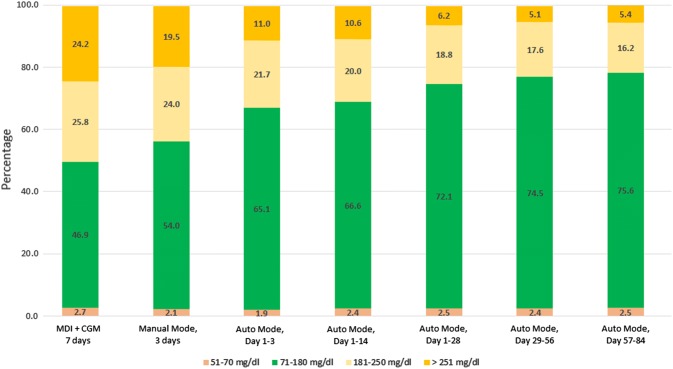
Time in ranges at baseline, during Manual Mode and Auto Mode periods. Values are shown as percentage spent in ranges during the interval. *MDI* multiple daily injections, *CGM* continuous glucose monitoring. Glucose values < 50 mg/dL are not shown on the graph: 0.3% in Manual Mode and Auto Mode period day 57–84, 0.4% in all other periods

Comparing different study periods, TIR (70–180 mg/dL) increased from MDI + CGM to Manual Mode (*p *= 0.010); Manual Mode to Auto Mode of 3 days (*p *= 0.003); Auto Mode of 3–28 days (*p *= 0.006); Auto Mode of 28–56 days (*p* = 0.698) and Auto Mode of 57–86 days (*p *= 0.147), as shown in Fig. 1. Time above range (> 181 mg/dL) decreased from MDI + CGM to Auto Mode of 57–84 days during the study (*p *= 0.021).

### HCL system usability

The total number of Auto Mode exits during the 3 months study phase was 5.1 ± 1.2 events per week, and significantly decreased between the first 2 weeks and the third month after enabling Auto Mode, from 8.4 ± 1.8 to 4.2 ± 0.9 events per week, respectively (p = 0.016) (Table 3). The mean number of daily calibrations also significantly decreased from 4.3 ± 1.2 to 3.4 ± 0.9 (*p *= 0.010). Infusion set and reservoir were changed on a regular basis every 2–3 days. Number of meals and carbohydrate intake did not differ from the beginning to the end of the study. ICR during initial Auto Mode period was made more aggressive regardless of the meal period compared with baseline and decreased from a median of 15 at the beginning to 12 at the end of the study (*p *= 0.002).

**Table 3:** HCL system characteristics during the Auto Mode period

	First 2 weeks of Auto Mode	Third month in Auto Mode	*p*
Sensor wear, %	89.8 ± 7.5 (92, 90–94)	92.4 ± 6.2 (94, 86–96)	0.038
Auto Mode usage, %	84.8 ± 11.9 (87, 77–97)	89.8 ± 7.4 (90, 84.5–96.5)	0.787
Calibration, *n* per day	4.3 ± 1.2 (3.8, 3.2–4.9)	3.4 ± 0.9 (3.4, 2.7–4.3)	0.010
Set change, *n* of days	2.5 ± 0.7 (3.0, 2–3)	2.8 ± 0.6 (3.0, 2.4–3.1)	0.269
Res change, *n* of days	2.9 ± 1.3 (2.2–2.3)	2.8 ± 0.6 (2.1–3.5)	0.315
Meals, *n* per day	4.4 ± 1.2 (4.4, 3.8–5.2)	4.6 ± 1.2 (4.4, 3.8–5.0)	0.331
Carbs, gram per day	213 ± 50 (205, 173–254)	219 ± 48 (221, 190–242)	0.704
ICR, gram	16.2 ± 5.7 (15, 12–20)	12.8 ± 4.8 (12, 10–16)	0.002
Active insulin time, h	3.6 ± 0.4 (3.3, 3.3–4.0)	3.5 ± 0.4 (3.5, 3–4)	0.155
Auto Mode exits per patient per week			
Total number^a^	8.4 ± 1.8	4.2 ± 0.9	0.016
No calibration^a^	3.3 ± 2.6	1.3 ± 1.5	0.004
High SG^a^	2.8 ± 1.7	1.2 ± 1.8	0.024
Insulin max delivery^a^	1.1 ± 0.9	0.5 ± 0.3	0.005

Values are shown as mean ± SD, median and interquartile ranges

*n* number, *Res* reservoir, *Carbs* carbohydrates, *ICR* insulin-to-carb ratio, *SG* sensor glucose, *Max* maximum. Only the top three Auto Mode exits reasons are shown

^a^Values shown as mean and SD

### Prior CGM versus no prior CGM users

HbA1c decreased in both prior CGM and no prior CGM users, from 8.1 ± 1.6% (65 ± 17.5 mmol/mol) to 6.7 ± 1.1 (50 ± 12 mmol/mol) (*p *= 0.021), and from 8.2 ± 0.9% (66 ± 9.8 mmol/mol) to 6.6 ± 1.6% (49 ± 17.5 mmol/mol) (*p *= 0.019), respectively. TIR improved from baseline to end of the study in both groups, reaching 75.2 ± 5.9 and 75.9 ± 7.3 during the third month in Auto Mode, respectively. No significant difference in HbA1c or TIR was found between these groups at the end of the study. We did not find significant difference in sensor wear, Auto Mode usage and numbers of calibrations between prior CGM and no prior CGM users at the end of the study.

### Safety

There was no event of severe hypoglycemia, diabetic ketoacidosis (DKA) or hospital admission during the study.

## Discussion

This study describes a structured initiation protocol of MiniMed 670G HCL system in individuals with type 1 diabetes on MDI, and its impact on glycemic control and safety for 3 months following Auto Mode activation. Totally, 79% of eligible candidates consented to the study and no attrition was observed during the 3-month follow-up. The patients will be monitored to determine long-term persistence. The high individual engagement demonstrated in our study was exemplified by high sensor usage and percent time in Auto Mode. Median sensor use was 92% of the time, which is similar to previous reports of 90.9% in children [17], of 88.6% in adolescent and of 93.1% in adults [19]. They spent a median of 89% of the time in Auto Mode, which is significantly higher compared to previous reports of medians of 80.6% in children [17], 75.8% in adolescents and 88.0% in adults [19] and 84.8% in another study [20]. Participants’ high engagement and no attrition can be an indicator of their motivation and satisfaction with the HCL system in improving their glycemic control.

The reduction in HbA1c, by a mean of 1.5% (16 mmol/mol), and in the SG, by a mean of 53 mg/dL (2.9 mmol/mol), observed in our study is greater than that previously reported in children [17] and adolescents, and similar to adults [19]. Totally, 70% of the participants reached HbA1c < 7.0% (53 mmol/mol) at the end of the study, which is the target established by the ADA and ISPAD guidelines for glycemic control in children [1, 2]. This is also significantly superior to what was observed in the previous MiniMed 670G pivotal trials.

The time spent within, below and above the target glycemic ranges has been defined as glycemic goals, beyond HbA1c [23, 24]. In our study, the median TIR (70–180 mg/dL) achieved in Auto Mode was 75.2%, which is similar to the previously reported medians of 73.5% [18] and 74.9% [19] in adults, but significantly higher than a median of 68.8% in adolescents [19] and 64.6% in children [17]. Time in range, time below range, and time above range observed in our study all achieved the desired clinical targets for CGM data interpretation, recently published by International Consensus on Time in Range [25]. These superior clinical outcomes, compared to the ones reported in previous studies of the MiniMed 670G system, which included experienced insulin pump users, were most probably driven by the high sensor and Auto Mode use. It is therefore possible that our specific initiation protocol, the parental/guardian involvement and supervision of the children, as well as the support and follow-up by the diabetes team, lead to the higher percentage of time spent in Auto Mode.

The significant TIR improvement was observed after only 3 days in Auto Mode, which indicates the effectiveness of our 10-days structured initiation protocol, as well as the rapid adaptation of the HCL system to the specific needs of the individual. After 3 days, TIR continuously improved over time until reaching a plateau after 2 months.

Our initiation HCL protocol for individuals on MDI allows to enable the Auto Mode feature in a 10-day period, which is significantly shorter compared to the HCL protocol group training for patient naïve to insulin pump therapy [20], where Auto Mode feature was enabled after 3–4 weeks from initial training. In the pivotal 670G studies in children [17], adolescent and adults [19], participants spent about 3 weeks in Manual Mode followed by 1–2 weeks of pump training, before Auto Mode was enabled. In our study, the short period of pump training and Manual Mode did not impact the glycemic control and safety of the participants; on the contrary, the superior clinical outcomes that were achieved in HbA1c (6.7 ± 0.5% (50 ± 5.5 mmol/mol)) and in median TIR (70–180 mg/dL) of 75.2% undermine the concept that closed-loop systems use require prior experience with diabetes-related technology to be effective and safe.

Additional insight from our experience is that modifying ICR by increasing the meal bolus dose by almost 20% during the first month of Auto Mode use is necessary when initiating individuals on HCL system from MDI regimens. Previous studies of HCL system reported similar findings [26]. This is dependent on the specifics of the algorithm [27–29] and may not be applicable for all HCL systems [30].

Both TDD and the percentage of daily basal insulin delivered slightly increased, which is similar to previously reported studies [17, 19]. Automated basal insulin delivery and ICR modifications, driving increase in meal bolus, effectively distributed the insulin delivery according to patients’ individual requirements, resulting in better control with minimal increase in total insulin dosages.

Similar results were achieved by participants with and without prior use of CGM, reinstating that the lack of prior experience with diabetes-related technology does not hinder the outcome with the MiniMed 670G system [31].

Auto Mode exits averaged 5.1 ± 1.2 per week in the study period, which is similar to previously published results of 5–6 exits [26] and 5.8 ± 1.6 exits [17], again attesting to the fact that prior insulin pump and/or CGM experience is not necessary for proper handling of the HCL system.

The main reasons for exiting Auto Mode were no calibration, high SG and maximum insulin delivery. The number of Auto Mode exits decreased from 8.4 ± 1.8 to 4.2 ± 0.9 events per week (*p *= 0.016), between the first 2 weeks and the third month on Auto Mode, respectively. In the same periods, the number of sensor calibrations decreased from 4.3 ± 1.2 to 3.4 ± 0.9 per day. This can indicate that the participants became more comfortable using the system over time, which is supported by the increased time spent in Auto Mode and the increased TIR achieved over time.

Importantly, the improved clinical outcomes observed in our study were achieved in a safe manner, with no events of DKA, or severe hypoglycemia, and with no hospital admission, similar to the MiniMed 670G pivotal trials.

There are several limitations of our study: absence of a control group, relatively short observation period (3 months) and different time periods in the evaluation of glycemic control. Additional limitation is that the protocol assessed only the minimal 72 h period in Manual Mode before turning Auto Mode on. Longer periods in Manual Mode might have had additional benefits. However, the objective of this study was to evaluate the protocol to initiate HCL system in individuals on MDI therapy, in comparison with the clinical outcomes reported in previous studies of the same HCL system.

## Conclusion

Children and adolescents with type 1 diabetes on MDI therapy can successfully initiate the HCL system, using a concise structured 10-day protocol, achieving better outcomes than in previous studies, where participants had previous experience with diabetes-related technology and longer initiation process. Further investigation on more varied population and ages should be performed to confirm these findings.
